# Restless Legs Syndrome and Poor Sleep Quality in Obese Children and Adolescents

**DOI:** 10.4274/jcrpe.5165

**Published:** 2018-05-18

**Authors:** Rıza Taner Baran, Müge Atar, Özgür Pirgon, Serkan Filiz, Meral Filiz

**Affiliations:** 1 University of Health Sciences, Antalya Training and Research Hospital, Clinic of Pediatric Endocrinology and Diabetes, Antalya, Turkey; 2 Süleyman Demirel University Faculty of Medicine, Department of Pediatric Endocrinology and Diabetes, Isparta, Turkey; 3 University of Health Sciences, Antalya Training and Research Hospital, Department of Pediatric Allergy, Antalya, Turkey; 4 University of Health Sciences, Antalya Training and Research Hospital, Department of Physical Therapy and Rehabilitation, Antalya, Turkey

**Keywords:** Obesity, restless legs syndrome, sleep quality, adolescent

## Abstract

**Objective:**

Adult epidemiological studies suggest that the rate of Restless Legs syndrome (RLS) in the general population may range from 5% to 15%. The aim of this study was to investigate the frequency of RLS in a community sample of obese adolescents aged 10-16 years and to assess the association with sleep quality and health-related glucose metabolism markers.

**Methods:**

The study group comprised 144 obese and overweight children aged 10-16 yearsand the control group consisted of 66 age-matched healthy children. The RLS Questionnaire devised by the International RLS Study and the Pittsburgh Sleep Quality Index (PSQI), where a score >5 indicates poor sleep quality, was used to assess sleep quality.

**Results:**

Mean body mass index (BMI) of the overweight/obese and control groups were 30.5±0.5 and 18.7±0.2, respectively. The frequency of RLS was higher in the obese group (21.7%) than the overweight (3.4%) and control (1.5%) (p<0.001) groups. The frequency of a poor PSQI score was significantly higher (p<0.001) in the obese group (37.3%) than the control group (24.2%). The obese with RLS group also had poorer sleep quality scores than the non-RLS obese group. Many symptoms of sleep disruption were more common in obese patients with RLS and RLS was independently correlated with a high PSQI score [odds ratio (OR): 2.25, confidence interval (Cl): 0.96-5.28, p<0.001)] and an increased BMI z-score (OR: 8.87, Cl: 2.04-38.61, p<0.001).

**Conclusion:**

RLS is common in obese children and may be associated with altered sleep quality. Obese children with RLS need to be assessed since they may need support to improve their sleep quality.

## What is already known on this topic?

Restless Legs syndrome (RLS) is a sensory-motor disorder characterized by feelings of discomfort, causing the desire to move the legs. RLS is also common in the pediatric population affecting 2-4% of school-aged children and adolescents. Sleep disturbance has been shown to be a commonly associated feature of RLS in the pediatric population.

## What this study adds?

This study demonstrated that the rate of Restless legs syndrome is higher in obese adolescents than in the general population. The rate is higher in patients with higher body mass index. Obese patients with RLS were found to have significantly more sleep-related symptoms.

## Introduction

Childhood obesity is associated with various adverse outcomes, such as poor academic performance, reduced psychological well-being, life-long obesity and cardiovascular diseases, which can all impair overall quality of life (1). Obese individuals are significantly more likely to report sleep disturbances (2). Today, there is increasing evidence indicating that sleep duration may be associated with obesity since sleeping plays a vital role in hormonal release, metabolic changes and lifestyle, which may result in obesity (3).

Restless Legs syndrome (RLS) is a sensory-motor disorder characterized by feelings of discomfort, causing the desire to move the legs (4). It manifests as an urge to move or the presence of unpleasant sensations in the extremities, symptoms that are worse with inactivity (while resting, sitting or lying down), which partially or wholly ease while moving the legs or walking, and are most severe at night (5,6). Currently, the pathophysiology of RLS is thought to be related with genetic predisposition, brain dopamine dysfunction and deficiencies in iron metabolism, although these factors have to date offered only a partial explanation (7).

RLS is usually associated with delayed sleep onset, difficulty in maintaining sleep, decreased total sleep time and reduced or absent slow-wave sleep (8). Sleep disturbance has been shown to be a commonly associated feature of pediatric RLS in population and clinic-based studies. Sleep disturbance is often the primary clinical complaint and more common in children with more severe RLS. Sleep disturbance is reported to be present in over 85% of pediatric patients with RLS (9,10,11). Six studies in adolescents reported that low sleep quality was negatively associated with body mass index (BMI) gain during the follow-up period (12).

The estimated prevalence of RLS has been reported to range between 4% and 29% in adults (13). RLS is less common in the pediatric population affecting 2-4% of school-aged children and adolescents (14,15). Some adult epidemiological studies have reported that BMI is associated with a higher likelihood of having RLS (16,17). However, unlike in adults, to date there have been no studies that have evaluated the prevalence of RLS and poor sleep quality in obese children. 

The main focus of this present analysis was to determine the frequencies of RLS and poor sleep quality in obese pubertal children using the new International RLS Study Group (IRLSSG) criteria. The secondary objective was to assess the impact of RLS on sleep quality and the relationship between glucose metabolism markers and lipids. It was hypothesized that the frequency of RLS would increase progressively as adiposity and insulin resistance (IR) increased, and that RLS would have a negative impact on sleep quality scores in children with obesity.

## Methods

### Subjects

A total of 115 obese and 29 overweight adolescents with a mean age of 13.1±1.7 years (range, 10-16 years), mean BMI of 30.5±0.5 were randomly recruited from among obese children who were admitted to the Pediatric Endocrinology Unit of Antalya Research Hospital between January and October 2017. The adolescents were grouped according to their BMI percentile values. Adolescents were excluded if they had a history of major illness, including type 1 or type 2 diabetes, were taking any medications, or had a condition known to influence body composition, insulin action, or insulin secretion (e.g. glucocorticoid therapy, hypothyroidism, Cushing’s disease). All subjects were in good health and had normal thyroid function. The control group consisted of 40 girls and 26 boys (mean age: 12.9±2.7 years, mean BMI of 18.7±0.2) who attended the hospital for minor illnesses such as common cold, conjunctivitis, or other similar condition. 

BMI was calculated as weight (in kilograms) divided by height (in meters squared). Patients with a BMI of ≥95^th^ percentile [BMI-standard deviation score (SDS) ≥1.64] according to reference curves for Turkish children were accepted as obese and BMI of 85-95^th^ percentile (BMI-SDS=1.04-1.64) as overweight (18). The pubertal development stages were assessed by a single pediatric endocrinologist using the criteria of Tanner stages. Staging for sexual maturation was >2 in all girls and boys (Tanner stages II–V) and considered as pubertal. The girls with menarche were excluded from the study. 

The study was approved by the Local Ethics Committee of the Antalya Research Hospital Institutional Review Board (approval number: 19.01.2017-2/17). Signed informed consent was obtained from each subject over age 12 years. Informed parental consent was obtained for all children regardless of age.

Plasma glucose, insulin levels and other parameters were determined in blood samples collected between 08.00 and 10.00 am, after fasting for 12 hours overnight. Glucose was determined by the glucose oxidase method. Serum insulin levels were measured with an immulite immunoassay system (Diagnostic Products, Los Angeles, CA). The homeostasis model assessment (HOMA) of IR was calculated as fasting insulin concentration (µU/mL) x fasting glucose concentration (mg/dL)/405. Iron and total iron binding capacity (TIBC) were studied using an Architect C8000 device (Abbott Laboratories, Abbott Park, IL, USA), ferritin on a DxI 600 device (Beckman-Coulter Inc., Pasadena, CA, USA) and hemoglobin on a Cell-Dyn Ruby device (Abbott Laboratories), all in accordance with the manufacturers’ instructions. Serum concentrations of total cholesterol, high-density lipoprotein cholesterol, and triglycerides were measured using routine enzymatic methods with an Olympus 2700 analyzer (Olympus Diagnostica GmbH, Hamburg, Germany). Low-density lipoprotein (LDL) cholesterol levels were calculated using the Friedewald equation.

### International Restless Legs Syndrome Study Group Rating (Symptom Severity) Scale

Pediatric or physical medicine residents asked face-to-face questions about the RLS diagnosis and severity based on the IRLSSG 2012 criteria. Pediatric diagnostic criteria are used for 10-12-year-old children while adult diagnosis criteria are used for 13-16-year-old children. Children were given a positive diagnosis of RLS if they met the following four criteria: 1) an urge to move due to uncomfortable sensations in the legs, 2) uncomfortable sensations are relieved by movement, 3) symptoms worsen during rest or inactivity, and 4) symptoms worsen in the evening (11).

### Pittsburgh Sleep Quality Index

This is a questionnaire assessing sleep quality as well as the presence and severity of sleep disorder. It includes seven components and 19 self-rated questions, assessing subjective sleep quality (e.g. ‘‘How would you rate your sleep quality overall?’’), sleep latency (e.g. ‘‘How long does it usually take you to fall asleep at night?’’), sleep duration (e.g., ‘‘How many hours of actual sleep do you get at night?’’), habitual sleep efficiency (time asleep vs total time in bed), sleep disorder (e.g. ‘‘How often do you have trouble sleeping because you wake up in the middle of the night or in the early morning?’’), use of sleeping medications and daytime dysfunction (e.g. ‘‘How often do you have trouble staying awake while driving, eating meals, or engaging in social activity?’’). All questions were rated between 0 and 3 points; 0: not during the past month, 1: less than once a week, 2: once or twice a week, 3: three or more times a week. In addition, sleep quality is rated as follows; 0: very good, 1: fairly good, 2: fairly bad, 3: very bad. Component scores are totalled to obtain a global score ranging from 0-21 points. Higher global scores indicate worse sleep quality, where a score >5 indicated poor sleep quality. The diagnostic sensitivity and specificity of the scale are 89.6% and 86.5%, respectively (19). The Turkish validation and reliability study was performed by Agargun et al (20) in 1996.

### Statistical Analysis

Mean and standard errors were used as descriptive statistics. Differences in the means of variables were tested using both parametric and non-parametric tests depending on the distribution of the variables. The Shapiro-Wilk W test was used to test for normality; p<0.05 was considered evidence for non-normality. Categorical variables across groups were compared using the chi-square test or Fisher’s exact test (if a cell number was five or less). Multivariable-adjusted logistic regression models were used to evaluate the association between the various risk factors and RLS and prevalent RLS. Odds ratios (ORs) and their corresponding 95% confidence intervals (CIs) were calculated. In the model evaluating the association between risk factors and RLS, RLS status was the dependent variable and independent variables were the various risk factors such as obesity, Pittsburgh Sleep Quality Index (PSQI) score, hemoglobin, ferritin, plasma glucose, plasma insulin and HOMA. All tests were two-sided; the level of statistical significance was at p<0.05. All analyses were performed with SPSS version 18.0 (SPSS Inc., Chicago, IL, USA).

## Results

The characteristics of the 210 adolescents in the study are shown in Table 1. No differences were found among the three groups with respect to mean age and gender. Obese and overweight subjects had slightly higher hemoglobin levels than control subjects and the obese group had elevated ferritin levels compared to the other two groups, although the ferritin and hemoglobin levels were within normal limits in all groups. There was no significant difference between the groups with respect to TIBC levels. Fasting glucose, fasting insulin, LDL cholesterol, triglycerides levels and HOMA values were increased in the obese group compared to the other two groups. The overweight group had higher fasting insulin and triglyceride levels than the control group but the other glucose metabolism markers such as HOMA values were similar.

### Frequency of Restless Legs Syndrome in Obese Children

**Figure 1:**
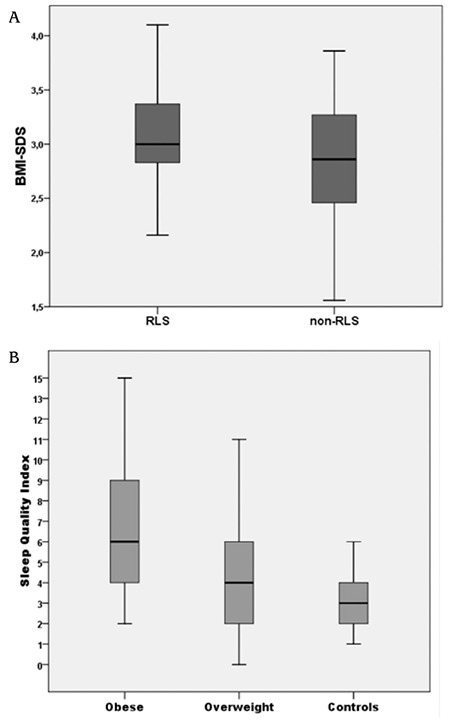
A) Boxplot for the distribution of body mass index - standard deviation score in obese children with restless legs syndrome and non- restless legs syndrome. B) Boxplot for the distribution of scores obtained through the Pittsburgh Sleep Quality Index used in children and adolescents according to their body mass index - standard deviation score BMI: body mass index, SDS: standard deviation score, RLS: restless legs syndrome

Overall, 12.8% of the cohort met the diagnostic criteria for RLS. Within the three study groups, the frequency of RLS was higher in the obese group (21.7%) than in the overweight (3.4%) and control groups (1.5%) (p<0.001). When compared to obese children diagnosed as RLS and non-RLS, BMI-SDS was higher in the obese children with RLS than the non-RLS obese children (3.04±0.46 vs 2.86±0.43, p<0.05) (Figure 1A).

### Sleep Characteristics in Obese Subjects with Restless Legs Syndrome

Poor sleep quality was found in 32.8% of the adolescents of the study group. The PSQI score was found to be higher in the obese group (5.45±0.2) than in the other two groups and the overweight group (4.21±0.5) had a significantly higher score than the control group (3.91±0.2) (Figure 1B). These differences were statistically significant and the obese and overweight groups had higher scores than the control group. Therefore, the frequency of poor sleep quality (>5 PSQI score) was higher in the obese group (37.3%) than in the control group (24.2%, p<0.001). Gender difference was not statistically significant among the groups.

When the obese patients with RLS and non-RLS were compared, the scores of subjective sleep quality (p=0.004), sleep latency (p<0.001) and sleep disorders (p<0.001) were significantly higher in the RLS obese subjects than in the non-RLS obese subjects, as reflected by the PSQI. The total PSQI score was significantly higher in obese subjects with RLS than in the non-RLS obese subjects (8.1±0.7 vs 4.5±0.2, p<0.001) (Table 2). When the poor and good scores for total PSQI scores were compared in all obese subjects, poor sleep quality subjects were found to have higher BMI and BMI-SDS values than those with good sleep quality (p=0.04). No significant differences with respect of concentrations of hemoglobin, plasma glucose, plasma insulin and HOMA valueswere found between obese subjects with poor (>5) and good PSQI (<5) (Table 3).

### Risk Factors for Restless Legs Syndrome

Multivariable logistic regression analysis revealed that increasing BMI was significantly associated with RLS when controlled for confounding factors. In this analysis, BMI-SDS (BMI-SDS >1.64; OR: 8.87, 95% CI: 2.04-38.61, p<0.001), and total PSQI scores (>5 score; OR: 2.25, CI: 0.96-5.28, p<0.001) were also independent significant risk factors for the incidence of RLS in adolescents. As was true for the full cohort, RLS in the obese group was independently and positively associated with age (OR: 0.83, CI: 0.35-1.98, p=0.02) and plasma glucose (OR: 3.68, CI: 0.86-15.72, p<0.001) but not with hemoglobin (OR: 1.98, CI: 0.25-15.8, p=0.87), ferritin (OR: 1.42, CI: 0.57-3.56, p=0.615), plasma insulin (OR: 1.29, CI: 0.51-3.27, p=0.343) and the HOMA value (OR: 3.02, CI: 1.32-6.90, p=0.086) (Table 4).

## Discussion

Firstly, the present study demonstrated that there is a significantly higher frequency of RLS in obese adolescents than in age-matched healthy control subjects (21.7% vs 1.5%). Secondly, obese patients with RLS were found to have many more sleep-related symptoms than those without RLS and RLS was found to be an independent predictor of poor sleep quality as reflected by the PSQI scores (OR: 2.25). RLS can be considered to be a common and clinically relevant sleep disorder in adolescents with obesity.

Although the pathophysiology of RLS is not yet fully understood, evidence exists for both iron/transferrin and dopaminergic abnormalities being factors in its etiology (10). Serum ferritin below 50 mcg/L was associated with increased severity of RLS in three adult studies (21,22,23). Recent pediatric case reports have also shown an improvement in RLS symptoms with oral iron therapy. However, iron deficiency is not common in all RLS sufferers and iron supplementation has shown variable success in RLS treatment (13,24,25). In this study, no relationships were found between RLS and serum levels of ferritin or hemoglobin, both of which have been reported to be related to the occurrence of RLS. However, it is possible that as the ferritin and TIBC levels were found within normal limits in all subjects, these were not detected as risk factors for RLS in the logistic analysis applied. In short, our findings suggest that low ferritin or iron deficiency has minimum or no impact on the development of RLS and that some undefined anemic condition might be required to increase the risk of the disorder. In most previous studies, anemia has been reported to be associated with increased risk for RLS, although approximately 70% of anemic adults do not develop RLS, and most patients with RLS do not have evidence of iron deficiency (7,26).

The diagnosis of idiopathic RLS is based on patient history as there are no physical characteristics or markers for the disorder. The disorder can be confirmed or ruled out on the basis of essential criteria defined by the IRLSSG. Two retrospective studies in adults have found the onset of RLS symptoms before the age of 20 years in approximately 40% of affected individuals (27,28). A large population-based prevalence study found RLS in 1.9% of children and 2% of adolescents in the United States and in the United Kingdom, respectively (29). More recently, a cross-sectional study carried out in Turkey estimated that the prevalence of RLS in non-obese children and adolescents was 2.9% (30). In the present study, the rate of RLS in the control group (1.5%) was found to be similar to the rate reported in previous studies on adolescents, while the frequency of RLS in obese patients was found to be significantly higher than that of the normal population (21.7%). Per et al (30) also reported that mean BMI value in adolescents was significantly higher in a group with RLS compared to those without RLS. These findings emphasize the importance of raising awareness of RLS among obese adolescents.

An association between obesity and a higher RLS prevalence has been observed in several adult studies (16,31,32). In a cross-sectional study including 1.803 men and women aged 18 years or older, each increase of 5 kg/m^2 ^BMI was associated with a 31% increased likelihood of having RLS (16). Several studies also suggest that RLS may be linked to key components of the metabolic syndrome, including diabetes, obesity and dyslipidemia. In an adult study, participants suffering from RLS were 4.7 times more likely to have impaired glucose tolerance and 8.5 times more likely to have elevated glycemia (fasting blood glucose >100 mg/dL) than the control group. Sleep disorders may have an association with decreased insulin sensitivity, independent of the association with adiposity (33,34). In the present study, obese patients had slightly elevated blood glucose levels but risk for RLS among the obese patients with elevated glucose levels or hyperinsulinemia was low. No correlation has been found among RLS and non-RLS adolescents for metabolic impairments such as glucose and insulin levels and HOMA, an IR marker.

A European primary-care study found that adult individuals whose RLS had a “high” negative impact on health had a significantly greater frequency of sleep disturbances (35). In another study by Picchietti et al (29), the sleep disorder rate was reported as 69.4% in adult patients with RLS. The excessive movements during sleep reported by obese patients with RLS may be secondary to the presence of periodic limb movements. In adults, leg movements are associated with 10-20% increases in heart rate and large elevations in blood pressure which begin at the time of leg movement onset and continue for 10-15 seconds afterwards (36). In the present study, RLS had a negative impact on sleep quality (OR: 2.25) in adolescents with obesity.

PSQI is a questionnaire which is useful in the evaluation of the quality and amount of sleep and the presence and severity of sleep disorders. In the present study, obese RLS patients were found to have elevated PSQI scores indicating poor sleep quality, especially in sleep latency, compared to non-RLS obese adolescents. There is also increasing evidence of an association between shortened sleep duration and/or poor sleep quality and obesity. In the current study, obesity was found to be significantly associated with an increased risk of developing RLS and poor sleep quality.

### Study Limitations

There are limitations to our study. Firstly, the number of cases with RLS was relatively small. Secondly, while the questionnaire was based on criteria established by the IRLSSG for children, these questionnaires are not fully validated in the pediatric population and can lead to misclassification. Despite these limitations, the present study has established that RLS is common in obese children and adolescents and is a significant cause of sleep-related symptoms.

## Conclusion

The results of this study demonstrated that the rate of RLS was higher in obese adolescents than in the general population and the rate increased as BMI values increased. It was also found that presence of RLS and a high BMI z-score, but not IR, have a significant impact on subjective sleep disturbances in obese patients. There is a clear need for further, randomized controlled RLS studies to better understand the metabolic response characteristics of the obese adolescent population.

## Figures and Tables

**Table 1:**
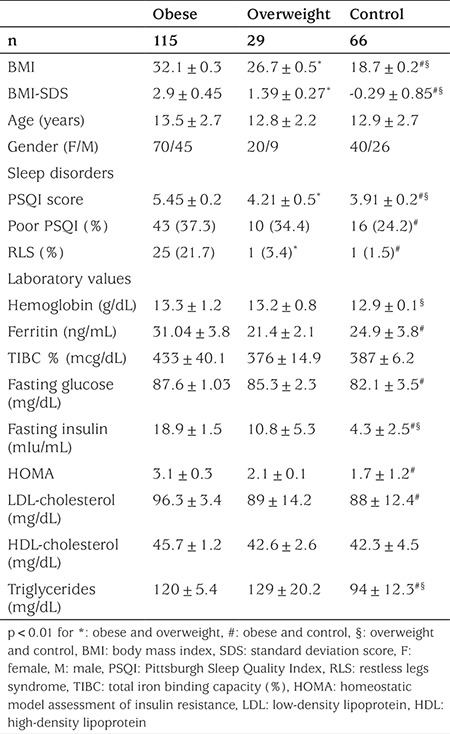
Characteristics of the study groups according to body mass index

**Table 2:**
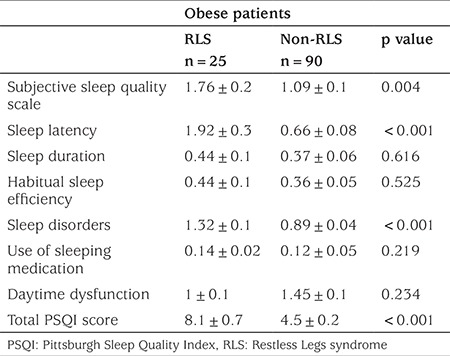
Comparison of sleep quality scores (Pittsburgh Sleep Quality Index) in obese children with restless legs syndrome and non- Restless Legs syndrome

**Table 3:**
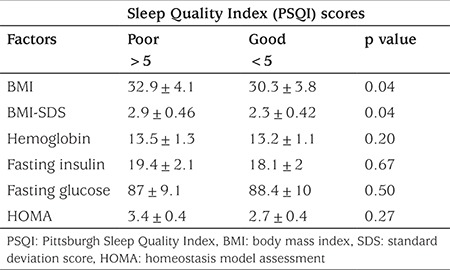
Sleep quality Index in obese children with Restless Legs syndrome (cut-off score for poor sleep quality was over 5 according to Pittsburgh Sleep Quality Index)

**Table 4:**
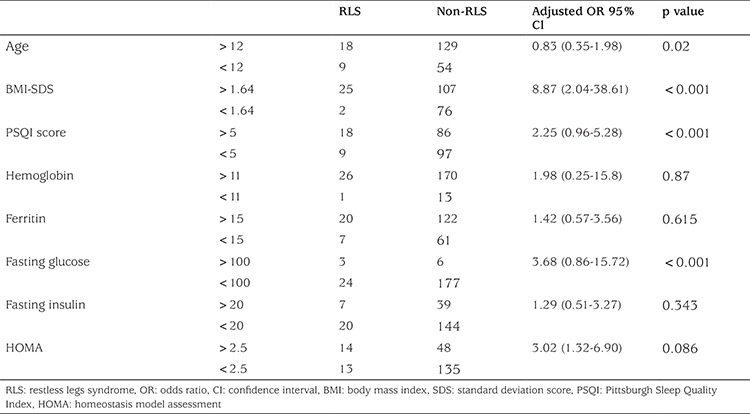
Relationships between risk factors and Restless Legs syndrome
